# Effect of nano-biodiesel blends on CI engine performance, emissions and combustion characteristics – Review

**DOI:** 10.1016/j.heliyon.2023.e21367

**Published:** 2023-10-30

**Authors:** Bahaaddein K.M. Mahgoub

**Affiliations:** Agricultural and Biological Engineering Department, Faculty of Engineering, University of Khartoum, Khartoum, Sudan

**Keywords:** Nano-biodiesel, Nano-additives, Performance, Emission, Combustion

## Abstract

The situation of greenhouse gas emissions with the effect of severe decline in oil reserves and the increase in energy demand are among the alarming periods to be faced. For this reason, biodiesel is considered as an alternative that can be used in the transport sector. The main reason for this research is to review the Nano-biodiesel application in a diesel engine for the purpose of improving the fuel properties, combustion efficiency of the fuel and reducing the emission. This article critically and in-depth examines the impact of the different nano-biodiesel blends available, as well as highlighting their quality variations and their impact on engine outputs. The impact of Nano-metallic additives such as Al_2_O_3_, CeO_2_, CNTs, CuO, GO, TiO_2_ and others on fuel quality and combustion was analyzed. Selected and critically archived articles were reviewed. Significant enhancement is reported for nanoparticle-based disperse test fuels in term of brake thermal efficiency. Maximum improvement in BTE of up to 24.7 % with Jatropha biodiesel (B20) blend with 50 ppm Al_2_O_3_ nanoparticles was reported. Maximum percentage of 25 % reduction in BSFC was reported for Acacia concinna biodiesel blend with 150 TiO_2_. The maximum percentages of emission level reduction were 70.94 % for HC, 80 % for CO and 30 % for NOx for methyl ester of waste cooking oil (B10) blend with 100 ppm TiO, JB20 blend with 20 ppm Al_2_O_3_ and B10 blend with combined 30 ppm Al_2_O_3_ and 30 ppm CeO, respectively. Therefore, it is recommended to further investigate several combinations of biodiesel blends with different metallic oxide nanoparticles at different concentrations. According to the assessment, future studies may focus on hybrid nanoparticles, which are composed of two or more nanoparticles in order to overcome the limitations of nanoparticles to one component, by improving the properties of the nanoparticle, achieving new properties that cannot be obtained by single nanoparticles.

## 1 Introduction

Fossil fuels play a crucial role in starting engines for electricity generation and domestic purposes [1], however, its combustion product and the increase in automobile use create negative effects on the environment. Therefore, people are promoting low-carbon living in today's world [2]. The consequences of using fossil fuels cause dangerously increased levels of unburned hydrocarbons (UBHC), carbon monoxide (CO) and nitrogen oxides (NOx), resulting in adverse ecological changes. In addition, flue gases cause air pollution and the released gases contribute greatly to the climate by trapping heat [3]. As a non-renewable resource, the oil as a fuel is running out and its price is unstable because of the increment in its use for power generation and transportation and the rapid increase in energy demands [3]. Therefore, the current scenario of fossil fuel depletion and rising fuel prices, the challenges facing the current global plan for low-carbon living and the identification of extent of energy and the challenges that is associated with the contamination caused by burning process of fossil fuels have motivated to research clean and sustainable energy sources. Furthermore, it has become very important to examine the possibility and outlook of harnessing technically attainable, readily obtainable and globally suitable renewable energy sources as an alternative fuel [4,5]. More important again, the environmental crises linked with the combustion of fossil oil have also alerted the researchers to look for strategies that can deal with the excessive worrying air pollution levels and its potential consequences. Among the various alternative fuels, biofuels have received much attention as the most desirable backup fuels for the field of transportation [6]. This is because these energy carriers are capable of powering machines by themselves while their harmful such as SO_X_, HC and CO are substantially lower than those of the fossil fuels.

### 1.1 Biodiesel

Biodiesel is one of the favorable alternative energy sources for the transport industry because of rapidly reducing petroleum reserves and increasing demand of energy, on the other hand, the risk of pollution [3]. The production and usage of biodiesel has only started in many countries. However, in many countries, it is not possible to mix biodiesel with higher concentrations. Many Asian countries have planned a long-term strategy for emissions reduction by using even low-concentration of blended biodiesel [7]. However, biodiesel is already used in many countries around the world and its advantages are obvious [8,9]. Biodiesel is a resource that continue to exist despite being consumed and it is widely produced using a several methods. The main production way of biodiesel is the esterification of animal, vegetable fats and used oils [[10], [11], [12]]. However, a significantly reduced cost of biodiesel production can be attained through ùtilization of waste as feedstock [13]. Biodiesel is similar to diesel in its properties, while it is distinguished by its oxygen content causing complete burning and therefore higher energy production (higher cetane number) [14]. Nevertheless, biodiesel combustion produce lower energy compared to diesel [15].

The main advantage of biodiesel is that, the engine does not require any changes maintaining almost the same performance produced by the existing diesel engine with almost zero emissions of HC, CO, and particulates specially under lean conditions [16]. Through intensive biodiesel fuel research [[17], [18], [19], [20]], it has been proved that biodiesel manufactured from various sources can be mixed with diesel to obtain a promising engine outputs. Some publications reported that shortening the engine life can be overcomed when fuel additives are added to biodiesel fuel [3]. Although biodiesel is clean combustion and sustainable biofuel, some issues such as thickening at lower temperatures, higher emissions and lower performance must be addressed scientifically [21]. Researchers reported undesirable effects from biodiesel blends [22], including the relatively low values of cloud and pour points, poor fuel injection, comparably low levels of calorific value and the high level of NO_x_ [23]. The main challenges of burning biodiesel are producing significant emissions in the form of NO_x_. Adding nanoparticles to biodiesel is a new way to reduce internal combustion engine (ICE) pollution without negatively affecting fuel efficiency or power output.

It should be noted that some reports have attributed the undesirable NO_x_ emissions and BSFC increase to the use of biodiesel [24]. The levels of BSFC and NOx emissions formed during the process of biodiesel combustion are generally affected by the grades of biodiesel blends versus diesel and the blend physio-chemical properties which is linked by the origin of biodiesel [25]. Maintaining the performance of CI engines with the lowest levels of exhaust emissions using biodiesel have become one of the hottest targets of the day. As a result, adding fuel additives is a new approach to achieve this goal [26]. Among the recent fuel additives for biodiesel, nanoparticles appeared as a promising new fuel additive to achieve the great improvement in performance and reduce emissions. Added nanoparticles to biodiesel shorten ignition delay, improve oxidation rate and reduce exhaust emissions. The oxygen atoms contained in the nanoparticles improve combustion. The nanoparticles disperse into the fuel and promote better air-fuel mixing and improved chemical reactivity during combustion, which improves performance, combustion and emissions quality. Adding nanoparticles to biodiesel reduces ignition delay makes it faster to start combustion.

### 1.2 Biofuel for improved engine performance and emission

In the future, ICE will stay the main source to provide energy for transportation [1]. CI engines are widely used as power/electrical sources in many applications because of the enhanced mechanical and thermal efficiency, lower fuel and energy usage, and remarkable sustainability. For this reason, diesel engines require improved combustion efficiency and lower emissions. In addition, the traditional fuels should be replaced by renewable energies [3]. Reliability, durability and fuel efficiency of diesel engines make them the commonly used type of engines in different sectors [27,28]. Diesel engines achieve high efficiency in an economically way by injecting a small amount of fuel into a highly compressed air. High particulate matter (PM) and NO_x_ emissions are still challenging engineering problems in today's diesel engines.

Biodiesel blended with conventional diesel is more suitable for CI engines without major engine changes [29]. In addition, these biodiesel blends have the same fuel thermos-physics and rating as diesel fuel contain more oxygen [30]. Although tremendous research on the combustion of biodiesel have been established, an increase in the level of NO_x_ emissions with a level of biodiesel in the mix out of 20 % is always a big barrier [31]. To manage the level of NO_x_, key process factors of engine operation including compression ratio (CR), fuel injection timing and pressure (FIT, FIP) are modified and monitored to improve the engine outputs when fueled by biodiesel [32]. Among all the engine setting, FIT is crucial because biodiesel is a much denser and less volatile fuel requiring long time and temperature of auto ignition to achieve cleaner burning process [33]. The advancement of FIT using biodiesel as a CI engine fuel has resulted in pre-combustion and higher in-cylinder temperature with less greenhouse gases (GHG) such as HC, CO, CO_2_ emissions [34]. With this, trade-offs were observed, such as higher in-cylinder and exhaust temperature [35], which increased NO_x_ emission levels [36]. Also, the delaying of the FIT affcets the reduction in burning process time and leads to a reduction in BTE [37], and NO_x_ emissions [38].

Various approaches to minimizing emissions formed outcome of the incomplete burning process of biodiesel blends have been explored. Some of them involve the use of some chemical composition of additives to promote the limited quality of biofuels [39]. New technology areas include fuel post-combustion emission control devices to help meet future emission standards [40]. The use of fuel additives was a cost-effective method, resulting in a reduction in particles below 2.5 lm [41]. Recent research indicates that organic and inorganic fuel additives may well solve the problem of NO_x_ emissions [42]. In this way, the performance of the engine can be achieved without changing the operating conditions of the engine and there is no need for exhaust gas after-treatment devices [43]. Fuel additives are mainly organometallic compounds that disperse entirely in the fuel. A metallic additive is added to diesel: to shorten the ID, as a stabilizer and antioxidant, and as a surfactant. Metal-based additives effectively minimize the level of emissions by production of hydroxyls when reacted with water, which promote oxidation of the soot or through the direct reaction of metals with the carbon atoms of the soot, thereby lowering the oxidation temperature. The metal inside the additives facilitate the soot oxidation when it is used after the combustion. Scientists and engineers are researching an alternative emissions control mechanism. The use of micron particles in fuel causes agglomeration and clusters to form in the fuel. Therefore, uniformly dispersing micro-particles in a fuel is a daunting task [44].

A method commonly used in recent times consists of nanoparticles whose size varies from 1 to 100 nm to solve this problem, because they are easily dispersible in liquid fuels [45]. Therefore, the researchers discovered that the nanoparticles compensate for the disadvantages of biodiesel. The metal nanoparticles behave as a catalyst for the burning process of hydrocarbon fuels cause complete fuel burning and decreased combustion products. This is because of the high surface-area-to-volume (Sa:Vol) ratio of nanoparticles which increase the adjoin between the blend and oxidant [46]. In addition, nanoparticles addition affect the time range of chemical transformation and cause a reduction of the ID time [47]. Furthermore, using of nanoparticles as additives to diesel could significantly shorten the evaporation time, which would shorten the effect of the ID, and therefore increase the probability of ignition of the mixture. This may prossibly be assigned to the enhanced ignition characteristics when nanoadditives used, leading to high catalytic activity because of the high Sa:Vol ratio.

## 2 Literature review

### 2.1 Nanoparticles application in CI engines

Scientists have made significant advances in nanoparticles application and related knowledge in recent years. Most nanoparticles are made from ceramics, metalics and polymers. Titanium, carbon, aluminium and iron are the widely used nanoparticles [48]. Many studies were peroformed on the combustion of liquids with carbon-based nano-additives [49]. Nanoadditives serve as carriers of secondary energy in liquids and enhance the burning process. Oxides of aluminium, cerium, copper, iron, cobalt, silver, silicon, graphene and titanium have recently been added to different blends. After comprehensive research, scientists have discovered that modifying the physicochemical properties of fuel is a more effective solution than making changes to the engine design to maintain the engine output at its highest levels with lower exhaust emissions [3]. Several options of metals-and metal-oxides-based nano-additives have been reported in the literature [50]. Nanoparticles of metal oxide are highly compatible with biofuels, as reported by Dreizin [51]. Metals such as iron (Fe), aluminium (Al), magnesium (Mg), manganese (Mn), silver (Ag), gold (Au), copper (Cu), boron (B), graphene, silica (Si), etc. and metal oxides such as aluminium oxide (Al_2_O_3_), cobalt oxide (Co_3_O_4_), CeO_2_, titanium oxide (TiO_2_), zinc oxide (ZnO), copper oxide (CuO) etc., are added in the micro or nano size to liquid fuels to improve its quality. Magnalium (Mg–Al) and Carbon nanotubes (CNTs) are examples of metal-based nanoparticles as well which increase fuel performance by modifying physiochemical properties.

### 2.2 Influence of nanoparticles on the physical and chemical properties of biodiesel fuel mixtures

This section analyzes the value of nano-additives dispersion into biodiesel and its impact on boosting the physical and chemical properties of nano-biodiesel blend. Nanofluids are a new type of solid-liquid composites consisting of solid particles having dimension of few nanometers dispersed into any base liquid [52]. Similarly, the nanoparticles can be added to biodiesel blends as base fuel. This new fuel is called nano-diesel-biodiesel. Fuel improvers are generally categorized as refining substances, dispersal system substances, and automotive output-enhancing substances. They are further divided into antioxidants, cetane enhancers, anti-knock mediums, anti-freeze mediums, stability enhancers, anti-corrosion ingredients, cold flow enhancers, fuel-based catalysts, anti-wear mediums, etc. [3]. Soudagar et al. [4,5] reported that different levels of nanoparticle are added to biodiesel to increase the oxygen concentration in the diesel particulate filter (DPF), improve the quality of liquid being stable in a broad range of conditions and the fuel physio-chemical properties. In addition, adding nanoparticles to biodiesel reduces the levels of exhaust emissions, enables the largest amount of fuel to come into contact with air atoms, improves viscosity index, reduces the ID time and improves interaction chemical-chemical.

Nano-biodiesel blend has proven to be strongly efficient and prospective fuel [53]. Because of the high amount of energy deposited into metal particles, high Sa:Vol ratio, elevated number of active centres needed for various reacting processes, increased rate of catalytic reaction, superior catalytic activity, the total calorific value of liquid fuel boost, which can increase the power output, which improves the engine performance. Reports have shown that fuel mixed with Al, B or carbon particles increases the probability of ignition and the onset of combustion at low temperatures compared to diesel, which reduces the ID [54]. A very important phenomenon that is involved in enhancing the combustion rate of fuel mixed with nanoparticles is the microdecomposition/explosion behaviour of blend droplets, which was first discovered by Takahashi et al. [55] for boron/JP-10 slurries. This behaviour has also been tested by several other studies involving masses of Al, B, Fe and carbon [56].

A research study by Javed et al. [57] revealed that nano-additives have the potential to raise the liquid blend volumetric energy density and thus tend to reduce the ignition lag by increasing the heat transfer rate. Mirzajanzadeh et al. [58] revealed that biofuel dispersed with nanoparticles would significantly lead to better fuel properties, especially its heating value due to the higher Sa:Vol ratio. This feature of nanomaterials helps better spraying, conversion of liquid fuel to gas and mixing with air for the burning process.

To date, some experimental tests were performed on utilization of nanoadditives into diesel and biodiesel mixtures and promising outputs were achieved. Nanoadditives including metals, metal oxides, carbon nanotubes (CNTs), graphene, organic components and hybrid nanomaterials are currently being investigated by researchers. Among the different types of nanoadditives, metal oxides are the most popular having a strong redox reaction with CO, HC and carbon atoms due to its oxygen content [59]. Hao et al. [60] reported that Al nanoadditives have an ability to extract oxygen and decrease the amount of energy required to complete the exothermic catalytic reactions with a significantly short induction time. Singh et al. [61] reported that, an increased ignition rate, ID and total combustion time can be achieved by the use of single-walled carbon nanotubes (SWCNTs) and multi-walled carbon nanotubes (MWCNTs).

The literature review proved that nanoadditives dispersed in liquid fuels have an impact on the improvement of its thermo-physical properties including flash point, fire point, kinematic viscosity, high Sa:Vol ratio, thermal conductivity and mass diffusivity [49]. Sarvestany et al. [62] observed that fuel density and viscosity can be slightly increased by the addition of nanoadditives. This can significantly improve the spraying process, rate of fuel to air mixing for burning process, and spray angle. Increased calorific value and cetane number was also ascertained when iron oxide nanomaterials were dispersed into the blends [63]. Arockiasamy et al. [63] reported an improved kinematic viscosity, density and calorific values when 30 ppm Al_2_O_3_ and CeO_2_were added to methyl ester of Jatropha (JME) blends. However, the addition of Ce with Jatropha biodiesel (JBD) did not make any change on the kinematic viscosity, density and calorific values, while the alumina added to JBD has shown an improvement in the fuel properties. Syed Aalam and Saravanan [64] noticed some improvement in the properties of a B20-Mahua biodiesel blend as well when Al nanoparticles are added to the mixture. Improvements in flash point, ID and cetane index were revealed by Prabu et al. [65] when nanoparticles are used as additives to biodiesel blends. Increased flash point and viscosity for nano-biodiesel was reported by Sajith et al. [66] when CeO_2_ nanoparticles are added at dispensing rate of 20, 40 and 60 ppm to biodiesel.

Fengsvanarak et al. [67] recorded a reduced kinematic viscosity and an increased cetane number of palm biodiesel when TiO_2_ nanoparticles are added. Ganesh et al. [68] reported increased flash point, volatility and viscosity of JBD when 10–20 nm CeO_2_ nanoadditives are dispersed into JBD. Noticeable decrease in the level of viscosity and calorific value was noticed by Shaafi and Velraj [69] when 100 mg/l of Al_2_O_3_ was mixed with diesel-soybean biodiesel-ethanol blend (D80SBD15E4S1), while the cetane number has increased.

Attia et al. [70] outlined the decrease in the kinematic viscosity and increase in density and cetane number of B20-Jojoba methyl ester blend when Al_2_O_3_ nanoparticles are added. Syed Aalam et al. [71] revealed the improvement in ZJME25 blend quality when Al_2_O_3_ nanoparticles are added. A blend of ZJME25 and 50 ppm Al_2_O_3_ provided better results than 25 ppm Al_2_O_3_ blend and boosted the values of flash point and cetane number. Increased flash point and density with reduced viscosity of canola methyl ester emulsion and diesel blend were reported by Anbarasu et al. [72] when Al_2_O_3_ nanoparticles are added. Basha and Anand [73] indicated that combining CNT with JBD provided a blend with better quality. However, it was reported that the flash point of JBD and CNT blend was lower than JBD and Al_2_O_3_ nanoparticles blend. Balaji and Cheralathan [74] reported an increase in the parameters of flash point, viscosity, calorific value and cetane number when CNTs are dispersed into biodiesel blend in different dosage rates.

Basha and Anand [75] examined the influence of CNTs dosage level on (JME) water emulsion blend properties namely the flash point, viscosity, density, calorific value and cetane number. The blend with the highest concentration of 100 ppm CNT recorded the lowest flash point, while an increased trend was noticed for the viscosity, density, calorific value and cetane number. The same conclusions were outlined by Karthikeyan et al. [76] when ZnO nanoparticled are added to different blends. Karthikeyan et al. [77] recorded an increase in the flash point, fire point, density and viscosity of B20-Grape seed oil methyl ester (GSOME) blend when ZnO nanoparticles are added. An increase in the calorific value and reduction in flash/fire point of B20-Pomoline Stearin Wax (PSW) blend were recorded when ZnO and CeO_2_ nanoparticles are added by Rao and Srinivas Rao [78] and Karthikeyan et al. [79].

Sunil Kumar Sharma et al. [80] examined the dispersion of CeO_2_ and CNT in JME with tire pyrolysis oil (TPO) blend and reported a reduction on the blend viscosity and density when CeO_2_ nanoparticles are added, while different results were recorded when using CNT, where there was a significant raise in density and viscosity. Gumus recorded an increase in the calorific value and cetane number of different biodiesel mix when nanoparticles are added. A reduction in sulfur content of the blend was also reported in some cases [81].

As discussed in previous studies, it can be concluded that the dispersion of CeO_2_, Al_2_O_3_, CNT, Al and GO nanomaterials into pure biodiesel decreases the values of flash point while increasing the values of viscosity and density [63,73,[82], [83], [84], [85]]. In addition, the calorific value of pure biodiesel increases when FBC, CNT, graphene and Al nanoparticles are added [74,82,85]. Furthermore, the utilization of ZnO nanoparticles increases the values of fuel flash point, viscosity, density and calorific value. While, Mn, Mg, Al nanoparticles in different mix decrease the value of flash point [64,86,87]. Moreover, a reduction in the blend flash and fire point was reported when fuel-supported catalysts are added [63,64,73,75].

### 2.3 Selection of dosage level of nanoparticles into fuel blend

Additives are metal-based substances readily soluble in blend and whose primary purpose is to enhance the blend quality by providing beneficial properties. The range of fuel additives amount is limited from 100 to several thousand ppm. The formation of new particles occurs as soon as the amount of additive exceeds a specific limit. The additive dosage limit depends only on the soot emission factor and on the type of engine used (turbo-charged and naturally aspirated engines show the same behaviour). Lower additive dosages were beneficial, while specific improvement was observed with higher additive concentrations [88]. All additives contain metal as the main element, which then appears as an oxide in the emissions, while the impact of additives on soot reduction is somewhat limited. Nanoparticles signify enhancement in the performance of the burning process, consequently augmenting the ratio of the useful work output to the engine heat input. Dispresing of nano-additives into fuels is performed to tackle the deficit in managing the level of NOx substances without a decrease in thermal efficiency. Therefore, the dosage level has to be controlled. Although metal and metal oxide nanoparticles are widely used in industry and many studies have been conducted on using nanoparticles as additives in fuels, there is still a gap in determining the dispersion level of nano-additive into fuel and the most favorable nanoparticles combination for improved performance outputs and less emissions of CI engines. Therefore, the application of nanoparticles to biodiesel blends is an exciting option that needs to be fully exploited. Also, using two different nanoparticles as fuel additives can achieve the integrated impact of the nanoparticles and improve CI engines' performance and emissions.

### 2.4 Nanofuels preparation and stability

Nanofluid is the product of the process of dispersing nanoparticles into a liquid in a uniform manner. Nanofuel is a suspension of nanoparticles (at least one dimension less than 100 nm) in a base fuel having superior properties, which improves the fuel combustion. The properties of this product, especially the stability are greatly affected by the type of nanoparticles that are added to the liquid and the preparation method [3]. Therefore, the selection of preparation technique, either one-step or two-step is important in order to have a good physical and chemical properties of nanofluids [89]. Some new techniques may be emerged by researchers for the preparation of nanofluids. Nanoparticles generally characterized by its high surface energy because of its large surface area. Therefore, they start agglomeration and sedimentation to form a micro-sized particle. The preceding investigations of blended biodiesel with nano-additives recorded a sintering, grouping and sedimentation of nanoparticles when metal-based are used as nanoadditives due to short-term stability [90]. Hence, researchers must carefully select a surfactant to enhance the stability. Table 1 summarizes the application of different metal and metal oxide nanoparticles that are added to various blends in CI engines, the effect of nanoparticle type, dosage level and preparation method on the nanofuel properties and stability.

**Table 1:** Summary of the effect of using metal nanoadditives, dosage level and preparation on fuel properties.

Fuel + Nano	Kinematic Viscosity (cSt)	Density (kg/m^3^)	Flash Point (°C)	Cetane Number	Calorific Value (J/kg)	Dosage Level (ppm)	Preparation Method	Ref
Diesel	2.84	835	72	–	44.62			[91]
POB20	3.15	842	92	–	42.59			
Diesel + INP	2.87	839	75	–	45.16	50		
POB20 + INP	3.18	844	94	–	43.19	50		
Diesel	2.20	835	48	–	42.3		Ultrasonicate apparatus at 50 Hz frequency for 30 min	[63]
Biodiesel	4.10	873	85	–	39.5			
JBD + Ce_2_O_3_	4.25	875	78	–	38.9	30		
JBD + Al_2_O_3_	4.30	876	76	–	38.7	30		
Diesel	3	815	56	47	42		Agitated for 30–45 min in an Ultrasonic shaker	[64]
MME	4.9	869	136	56	39.35			
MME20	3.4	826	76	49	41.62			
MME20 + ANP	3.37	827.5	71	49.5	41.66	50		
MME20 + ANP	3.33	829	65	51	41.69	100		
Diesel	2.54	833	50	52	–		Agitated for 30–45 min in an Ultrasonic shaker.	[71]
ZJME25	3.56	846	56	56	–			
ZJME25 + Al_2_O_3_	3.39	849	57	57	–	25		
ZJME25 + Al_2_O_3_	3.17	853	58	58	–	50		
Diesel	2.61	–	–	57	44.7		Two-step method was used. Ultrasonicator was used to disperse the particles.	[69]
SBD	4.78	–	–	49	41.2			
D80SBD20	3.70	–	–	42	43			
D80SBD15E4S1+Al_2_O_3_	3.37	–	–	52	42.59	100		
JME	5.05	895	85	53	38.88		Mechanical agitator, ultrasonicator and a reactor vessel were used.	[75]
JME2S5W	5.40	899.8	140	51	37.05			
JME2S5W25CNT	5.43	897.2	130	54	37.28	25		
JME2S5W50CNT	4.76	897.8	125	55	37.35	50		
JME2S5W100CNT	5.91	899.4	122	56	37.85	100		
B20 (GSO biodiesel)	5.55	841	38	–	37.02		The blends were prepared with the aid of an ultrasonicator	[77]
B20ZnO	5.82	842	40.3	–	38.12	50		
B20ZnO	5.88	844	42.1	–	38.75	100		
B20 (PSW)	3.10	834	46	57	44.07		An ultrasonicator was used.	[79]
B20ZnO	3.10	833	47	58	44.33	50		
B20ZnO	3.10	832	47	58	44.30	100		
POME	–	–	–	–	–		Ultrasonicate with 50 % power and 0.7 s cycle for 30 min	[92]
POME + Al_2_O_3_	–	–	–	–	–	50		
POME + SiO_2_	–	–	–	–	–	50		
B20	4.71	0.81	157	52	43590			[93]
B20 + CuO_2_	5.1776	0.8316	157.5	54.5	44606.5	25		
B20 + CuO_2_	5.6451	0.8317	176	54.75	45623	50		
B20 + CuO_2_	5.6669	0.8318	176.5	55	49524	75		
B20 + CuO_2_	5.6888	0.9319	177	55.5	46219	100		
JPD	5.25	895	85	53	38.88			[73]
JPD + Al	5.31	896	84	54	39.22	25		
JPD + Al	5.35	897	82	56	39.53	50		
JPD + CNT	5.29	895.5	83	55	39.5	25		
JPD + CNT	5.33	897.9	81	57	39.78	50		
JPD + Al + CNT	5.36	895.2	81	57	39.99	25 + 25		
Diesel	2.4	0.82	48	48	42957		A water bath shaker at 60 °C for a reaction time of 60 min was used.	[94]
CSBD	3.7	0.75	140	51	37964			
CSBD + TiO_2_	3.3	0.72	141	52	38067	50		
CSBD + TiO_2_	3.5	0.74	141	53	38150	100		
Diesel	2.93	835.4	92.5	54.919	–		Thermomechanical mixer was used.	[95]
B10	3.17	844.6	>100	51.147	–			
B10 + TiO_2_	3.19	844.7	>100	52.134	–	100		
B10 + Al_2_O_3_	3.19	844.7	>100	51.983	–	100		
B10 +SiO_2_	3.19	844.7	>100	51.676	–	100		
HOME	5.60	875	187	40	36.10		An ultrasonicator was used.	[82]
HOME + Ag	5.80	895	160	–	35	25		
HOME + Ag	5.80	900	158	–	35.50	50		
JME90TPO10	6.38	868.7	–	–	–		The mixture was stirred vigorously for 60 min with the help of a tip sonicator, maintaining the sample at 40 °C temperature.	[80]
JME90TPO10CeO_2_	6.39	868.3	–	–	–	100		
JME90TPO10CNT	5.24	872.6	–	–	–	100		
JME80TPO20	6.36	874.1	–	–	–			
JME80TPO20CeO_2_	6.40	873.5	–	–	–	100		
JME80TPO20CNT	5.35	878.1	–	–	–	100		
JME70TPO30	6.47	880.4	–	–	–			
JME70TPO30CeO_2_	6.38	880.3	–	–	–	100		
JME70TPO30CNT	5.29	881.8	–	–	–	100		
Diesel	2.60	835	73	47	43.76			[96]
B25 (Pomace oil) + Mn	2.90	828	76	49	43.54	12 μmol/l		
B60 (Tall oil)	5.30	–	88	–	–			[87]
B60–8Mn	4.80	–	81	–	–	8 μmol/l		
B60–12Mn	4.30	–	80.5	–	–	12 μmol/l		
B60–8Ni	4.90	–	85	–	–	8 μmol/l		
B60–12Ni	4.80	–	79	–	–	12 μmol/l		
HOME	5.6	880	170	–	36.016	25		[84]
HOME + GP	5.8	895	160	–	35.00	25		
HOME + GP	5.8	900	158	–	35.50			
Diesel	4.24	835.0	–	46	45.72			[97]
Biodiesel + Ag	4.36	854.7	–	47	46.44	40		
Biodiesel + Ag	4.40	855.3	–	48	46.68	80		
Biodiesel + Ag	4.49	858.8	–	50	46.92	120		
Biodiesel + CNT	4.74	879.9	–	57	47.12	40		
Biodiesel + CNT	4.82	884.3	–	59	48.02	80		
Biodiesel + CNT	4.91	891.6	–	61	48.68	120		
Diesel	3.12	825	53	53	43.57			[98]
Neem oil	6.77	875	172	172	36.5			
BN20	3.74	828	65	65	41.9			
BN20 + Ce_2_O_3_	3.71	830	66	66	41.94			
PSBD	4.28	844	140	–	37.510			[99]
PSBD + 10 nm AgO	4.12	834	138	–	37.854	5		
PSBD* 10 nm AgO	3.98	819	136	–	38.012	10		
PSBD + 20 nm AgO	3.86	804	134	–	38.352	5		
PSBD + 20 nm AgO	3.71	797	132	–	38.542	5		
Diesel	2.4	820	50	–	42.412			
Diesel	2.3	839.5	75	51	44.50			[74]
Methyl ester of neem oil (MENO)	4.27	890	180	53	40.67			
MENO + CNT	4.28	889	181	53	40.92	100		
MENO + CNT	4.28	889	181	54	40.92	200		
MENO + CNT	4.29	888	182	54	40.92	300		
MENO + CNT	4.29	888	182	55	40.93	400		
Calophyllum Inophyllum biodiesel (CB) + TiO_2_							a magnetic stirrer	[100]
Diesel + Fe						50	ultra-sonication process	[91]
PB20 + Fe						50		
Diesel + Ailanthus Altissima biodiesel + GO						30	ultrasonic processor for 20 min	[101]
						60		
						90		

To achieve a stable nanofluidliquid, nanoparticles must evolve into surface modification. A dispersant is often used to prevent particle agglomeration during nanofluid formation. Ammonium Polymethacrylate and sodium silicate are well-known dispersants for water-based Nanofluids. The stability of nanofluids affects the thermo-physical properties of the nanofluids so it is very important to study the stability. Various methods including sedimentation method, zeta potential analysis, UV–vis spectrophotometer, scanning electron microscope, method equilibrium method dynamic light scattering are to assess the stability of nanofluids. The sedimentation method is the best method for evaluating the stability of nanofluids over time. A stable nanofluid is formed when the concentration of supernatant particles does not change with elapsed time. In this method, sedimentation photos of nanofluids are taken with a camera in test tubes and the stability of the nanofluid is observed. The disadvantage of this method is the long observation time to control particle precipitation. The zeta potential is the potential divergence among the bulk liquid and the stationary liquid layer attached to the nanoparticles. The high zeta potential (negative or positive) indicates a stable suspension system, while the nanofluids of lower zeta potential lead to faster sedimentation of nanoparticles [102]. Another effective way to predict the stability of suspension is spectral absorption analysis. UV–vis spectroscopy measures the absorption of light incident on nanoparticles. Zhu et al. [103] observed experimentally that the absorption is directly relative to certain number of nanoparticles in suspension, therefore, a higher one results in stable nanofluids.

## 3 Effect of biodiesel blended nanoadditives on CI engines performance, emissions and combustion

Improving the combustion of biodiesel blends of CI engines through the addition of nanoadditives for better engine outputs and lower levels of emission is an important topic needs more research [5]. The first try to use nanoadditives was carried out by Boutonnet et al. [104] with the use of monodispersed metal particles such as Platinum (Pt), Palladium (Pd), Rhodium (Rh) and Ir in the form of micro-emulsion. Then other metal nanoparticles were investigated by researchers for the purpose of enhanced engine stability, burning process and its products characteristics through the improvement of fuel blend properties and solubility [105]. All nano-additives selected by researchers and blended with different types of biodiesels to run CI engines are compiled, reviewed and discussed in this study to find out how mixing biodiesel blends with a few grams of nanoparticlescan improve combustion characteristics and performance parameters and reduce exhaust emissions.

### 3.1 Metal based additives

The properties of fuel burning process might be improved through adding metals or/and metal oxides nanoparticles in the micro or nano size range in ppm or weight percent ratios to the fuel.

#### 3.1.1 Effect of Al_2_O_3_ nanoadditives on biodiesel blends combustion

**Fig. 1:**
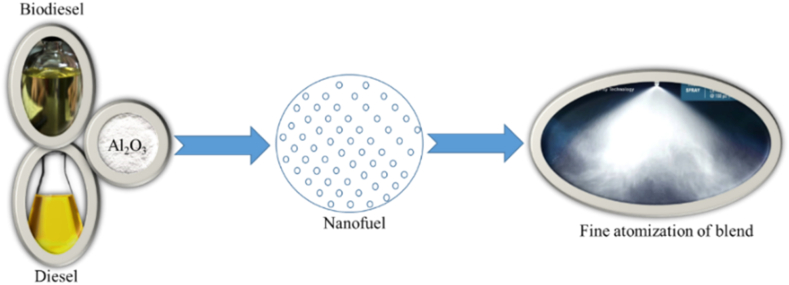
Atomization of nanoparticles dispersed test fuel.

Among the options available for harmful emissions levels reduction, fuel-based catalysts are recently gaining attention because of the advantages of fuel efficiency while controlling emissions, harmful GHGs, nitrogen oxides and particulate matter. Al_2_O_3_ is given great attention among the metal oxide nanoparticles due to its high Sa:Vol ratio. It has the advantage of improving atomization, allowing more fuel to react with oxygen, improving rapid evaporation and providing a better air-fuel mixing environment. Al_2_O_3_ nanoparticles decompose into Al_2_O and O at higher in-cylinder temperatures. The “Al_2_O″ molecule is considered a highly unstable compound at higher in-cylinder temperatures in the combustion chamber, which can further dissociate into 2Al and ½ O_2_ [106]. Equations below show the excess oxygen molecule joins the CO to turn into a CO_2_ molecule. Fig. 1 shows how the atomization process of biodiesel blend occur when Al_2_O_3_ nanoparticles are dispersed.

(1) $${Al}_{2}O_{3}\to {Al}_{2}O+2O$$

(2) $${Al}_{2}O\to 2Al+\frac{1}{2}O_{2}$$

(3) $$O+CO\to {CO}_{2}$$

Previous research reported that when the Al_2_O_3_ nanoparticles are dipersed into biodiesel it affects the mixture properties, engine performance, and emissions. Some previous studies [95,[107], [108], [109]] revealed that adding Al_2_O_3_ nanoparticles to biodiesel achieved better engine performance and emission results compared to other combinations, while higher emissions of CO_2_ and NOx were reported. Vennu and Appavu [105] reported that the use of Polanga Biodiesel (PBD) in a CI engine when Al_2_O_3_ nanoparticles are dispersed has increased the BTE by 4.21 % and 6.58 % with 25 ppm and 50 ppm concentrations. Similarly, the BSFC was reduced by 7.38 % when nanoparticles are added. Subsequently, a reduction of up to 30.32 % for HC, 31.49 % for CO and 37.34 % for smoke opacity emissions was reported. Raju et al. [110] revealed that the peak BTE detected from the test findings of the methyl ester of tamarind seed oil (TSME20) and ANP60 blend in single cylinder CI engine was 1.56 % more than diesel at maximum engine load. Al_2_O_3_ nanoparticles dispersed in the TSME20 biodiesel blend achieved 87.4 % smoke opacity and 56.6 % CO emission lower than diesel when the engine is fully loaded. Reforming the TSME blend by the addition of nanoparticle recorded a 24–68 % decrease in the level of UHC emissions and 7–9% in the level of NO_x_ emissions. Soudagar et al. [4] revealed that using of a HOME2040 blend on a CI engine showed an improvement in BTE by 10.57 %, decreased BSFC of 11.65 % and decreased levels of HC, CO, and smoke by 26.72 %, 48.43 %, and 22.84 % respectively, while an increase of up to 11.27 % of NOx emissions was recorded. The experimental study by Gurusala and Selvan [111] reported a smoke reduction of 52.8 % for the waste chicken fat biodiesel (B40) blend when 50 ppm of Al_2_O_3_ nanoparticles are added at fully load engine condition and reached about 65 % compared to pure diesel. Aalam and Saravanan [89] experimentally revealed that the use of MME20 biodiesel blended has substantially improved the BTE and marginally reduced the emissions when Al_2_O_3_ nanoparticles are added. The levels of HC and CO were decreased by about 26.04 % and 48 %. The experimental study by Patel Kumar [112] showed 3.85 % improvement in brake power and 24.7 % in BTE when 0.5 g/l of Al_2_O_3_ nanoparticles were added to JBD20. Further increase in Al_2_O_3_ nanoparticles at 0.75 g/l and 1.0 g/l into B20 caused a decrease in the BTE by 13.56 % and 26.39 %, respectively. The study recommended a concentration of 0.4592 g/l into B20 as the optimum value for an improved engine outputs and reduced knocking phenomenon caused due to the presence of the biodiesel. The analysis of the results obtained by Aalam et al. [71] showed that adding Al_2_O_3_ nanoparticles at 50 ppm to ZJME25 blend resulted in a considerable decrease of about 6 % in BSFC with a higher BTE of 2.5 % than that of the base fuel. It was also reported that, the smoke density of ZJME25 recorded about 15–20 % reduction when Al_2_O_3_ nanoparticles are added, especially at high engine load conditions. Hossain and Husain [113] reported that the BTE of JBD with 100 ppm Al_2_O_3_ nanoparticles fuel was higher than the BTE recorded of pure diesel by about 3 % while it was quite similar to that of pure JBD. At fully loaded engine condition, the brake specific energy consumption (BSEC) of JBD with 100 ppm Al_2_O_3_ nanoparticles fuel was 4 % higher and 6 % lower than that for pure JBD and pure diesel, respectively. NOx emissions were 4 % lower when JBD mixed with 100 ppm Al_2_O_3_ nanoparticles compared to JBD. The levels of UHC and smoke emissions were significantly reduced when 100 ppm Al_2_O_3_ nanoparticles are added to JBD blend instead of pure JBD or pure diesel fuels.

The experimental investigation carried out by El-Seesy [114] reported that the greatest mechanical output was acquired at an Al_2_O_3_ dosage level of 40 mg/l in addition to Jojoba biodiesel (JB20) blend, where BSFC got decreased by 12 %, BTE got enhanced by 15 %, P_max_ got raised by 4.5 %, dP/dθ_max_ by 4 %, and dQg/dθ_max_ by 4 %. The peak mitigation in levels of exhaust products was captured at a dosage of 20 mg/l, where NO_X_ was diminished by 70 %, CO by 80 %, UHC by 60 % and smoke opacity by 35 %. The suggested dosage level of Al_2_O_3_ to significantly improve engine outputs and emissions were 30 mg/l.

**Fig. 2:**
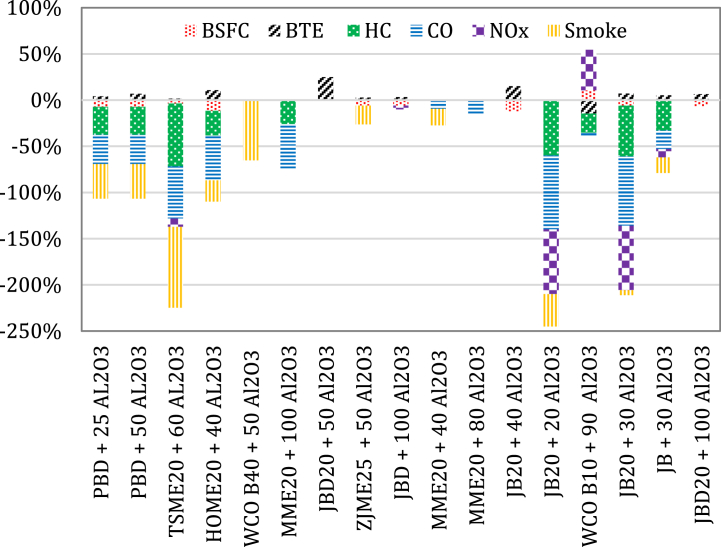
The average percent change in engine outputs for biodiesel blends with Al_2_O_3_ nanoparticles.

The experimental investigation carried out by Hosseini et al. [115] reported a change of +5.36 % for torque, +5.36 % for power, +10.63 % for SFC, −14.66 % for BTE and +5.80 % for EGT when 90 ppm Al_2_O_3_ nanoparticles were added to WCO biodiesel (B10) blend compared to that of diesel. CO and UHC levels got decreased by up to 2.94 % and 20.56 %, correspondingly; however, the level of NO_x_ emissions increased by 43.61 %. The dosage of 30 mg/l Al_2_O_3_ nanoadditives was determined by Attia et al. [70] as the appropriate concentration to mix with JB20 blend corresponding to optimum engine performance. At this dose, the total BSFC was diminished by approximately 6 %, the engine BTE got enhanced by about 7 %, and reduction by up to 70 % for NO_X_, 75 % for CO 5 % for smoke opacity, and 55 % for UHC was recorded compared to the corresponding values obtained when only JB20 fuel blend was used. A 5 % increase in BTE was observed by Arockiasamy and Anand [88] for the fuel of JBD blend with 30 ppm Al_2_O_3_ nanoparticles compared to pure biodiesel. A decrease of up to 9 % for NO, 33 % for UHC, 20 % for CO and 17 % for smoke opacity were also recorded than that of pure biodiesel. Reduced SFC, increased BTE and decreased EGT by 6.5, 6.5 and 27 %, respectively, were achieved by Gad and Jayaraj [116] when JBD20 blended with 100 ppm Al_2_O_3_ was used rather than JBD20 to run a CI engine. Fig. 2 summarizes the engine outputs when Al_2_O_3_ nanoparticles are added to different biodiesel blends.

#### 3.1.2 Effect of CeO_2_ nanoadditives on biodiesel blends combustion

CeO_2_ nanoparticles serve as a catalyst in reducing toxic gases throughout the burning process of hydrocarbon fuels and improving fuel economy. The amount of oxygen supplied and reversible from the gas phase is known as CERIA's OSC, and the presence of CERIA in the fuel helps to regenerate the diesel particulate filter at lower temperatures. CeO_2_ acts as an oxygen donor catalyst causes the reduction of NOx emission by providing the suitable oxygen amount for CO oxidation and oxygen absorbtion. The activation energy of CeO_2_ burns the carbon store in the engine cylinder at wall surface temperature. This reduce the level of HC by stopping the sedimentation of non-polar compounds on the cylinder wall surfcee. The key to using CeO_2_ for catalytic purposes is the low redox potential between the Ce_3_+ and Ce_4_+ ions (1.7 V) which make the following reaction easily occur in the exhaust [117].

(4) $$2CeO_{2}↔{Ce}_{2}O_{3}+\frac{1}{2}O_{2}$$

Hydrocarbon combustion:

(5) $$\left(2x+y\right)CeO_{2}+C_{x}H_{y}\to \left\{\frac{\left(2x+y\right)}{2}\right\}{Ce}_{2}O_{3}+\frac{x}{2}{CO}_{2}+\frac{y}{2}H_{2}O$$

Soot burning:

(6) $$4CeO_{2}+C_{soot}\to 2{Ce}_{2}O_{3}+{CO}_{2}$$

Cerous oxide (Ce_2_O_3_) created from the process of hydrocarbon oxidation gets re-oxidized to CeO_2_ via the diminution of nitrogen oxide.

(7) $${Ce}_{2}O_{3}+NO\to 2CeO_{2}+\frac{1}{2}N_{2}$$

Different studies [80,[118], [119], [120], [121]] reported an enhancement in the engine outputs and a noticeable reduction in the level of combustion products from exhaust when CeO_2_ nanoparticles were added different types of biodiesel blends. The consequences of using as additives in lemongrass oil (LGO) emulsion fuel have been experimentally studied. Based on the experimental investigations by Annamalai et al. [122], the BTE of lemongrass oil (LGO) nanoemulsion with CeO_2_ nanoparticles increased by 17.02 % and fall by 7.7 % contrasted to LGO and diesel fuels, respectively, at full engine load. The LGO nanoemulsion showed up to 5 % less BSEC than LGO and diesel fuel. LGO nanoemulsion fuel had an UHC and CO emissions reduction of 35.5 %, 16.03 % and 15.69 %, 26 %, compared with LGO and pure diesel fuel, respectively. NO_X_ emissions reduction for LGO nano emulsion fuel 24.8 % and 20.3 % than that of pure LGO and pure diesel fuel was reported. LGO nanoemulsion fuel had 6.4 % and 19.8 % reduction of smoke in comparison to LGO emulsion and pure diesel fuel. Sathiyamoorthi et al. [98] revealed that higher BSFC and a higher BTE of up to 2.8 % were recorded when CeO_2_ nanoparticles are added to BN20 blend. Also, emissions were reduced by up to 8.4 % for NOx, 4.4 % for smoke, 2.7 % for HC and 3.4 % for CO. A higher P_max_ of up to 8.1 % and a higher heat release rate (HRR) of up to 18 % were recorded during the BN20 fuel mode mixed with CeO_2_ nanoparticles.

**Fig. 3:**
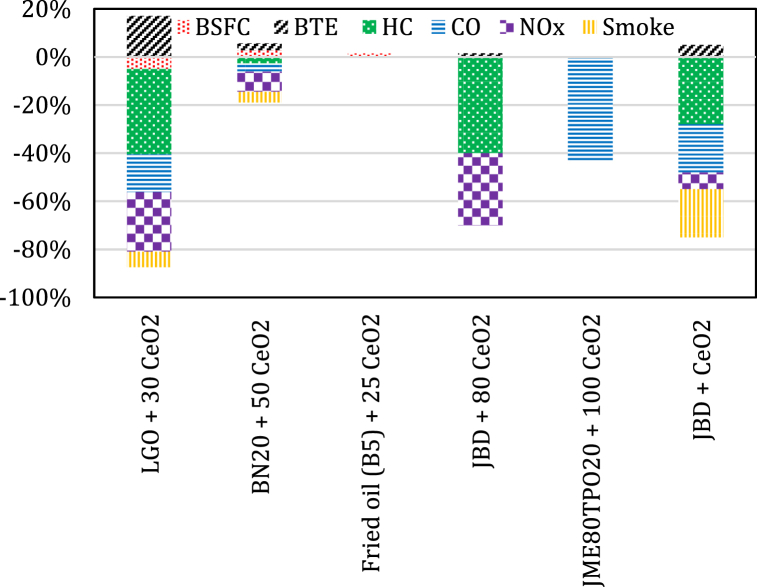
The average percent change in engine outputs for biodiesel blends with CeO_2_ nanoparticles.

The experimental investigation performed by Sajith et al. [66] revealed that an increased BTE by a maximum of 1.5 % was achieved when 20–80 ppm dosage level of CeO_2_ nanoparticles are added to biodiesel and the peak improvement in engine outputs are gained at the dosage of 80 ppm. The emissions of HC got reduced by about 25 %–40 % for dosage levels between 40 and 80 ppm of the CeO_2_ nanoparticles. Also, a reduction of up to 30 % was recorded when 80 ppm of CeO_2_ nanoparticle are added. The experimental results obtained by Sharma et al. [80] revealed a significant reduction of CO emissions by approximately 43.11 % for JME80TPO20CeO_2_100 than that of diesel. The BTE was about 24.25 % when the engine is fully loaded, which is much exceeding that of the JME80TPO20. The JMETPOCeO_2_ blend produced lower levels of NOx, HC and smoke density in the exhaust emissions than that of JMETPO blends. Arockiasamy and Anand [63] reported a 5 % improvement in BTE with a percent reduction of up to 7 % in NOx emission, 20 % in smoke opacity, 28 % in UHC and 20 % in CO for JBD blend with CeO_2_ nanoparticles. Fig. 3 summarizes engine outputs when CeO_2_ nanoparticles are added to different biodiesel blends.

#### 3.1.3 Effect of CuO and CuO_2_ nanoadditives on biodiesel blends combustion

**Fig. 4:**
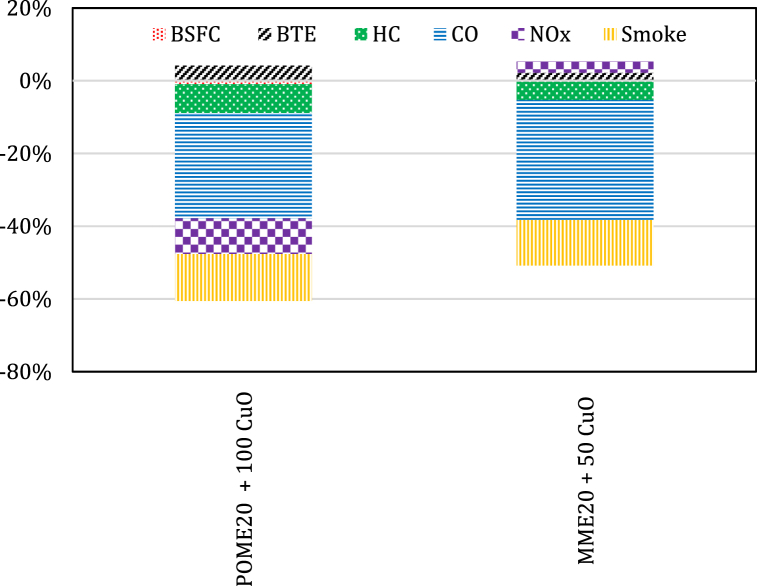
The average percent change in engine outputs for biodiesel blends with CuO nanoparticles.

Considerable attention was received by CuO_2_ nanoparticles as catalysts that is characterized by its activity with an inexpensive cost for various reactions of gas-solid. This high catalytic activity of CuO_2_ comes from its reducibility and surface reactivity. In addition, it is a popular choice of oxygen carrier in the literature due to its low cost and exothermic reduction reactions both with carbon and CH_4_. Therefore, CuO_2_ nanoparticles are useful as a catalyst for processing aromatic hydrocarbons. Dharmaprabhakaran et al. [123] revealed that adding CuO_2_ nanoparticles to Botryococcus Braunii Algae (BBMAE) blend for combustion in a CI engine provided higher BTE and reduced the BSFC. Kalaimurugan et al. [124] reported that the neochloris oleoabundans methyl ester-diesel blend (B20) added to the CuO_2_ nanoparticles had a great improvement in BTE, EGT and BSFC compared to B20. It also showed the highest Pmax, dP/dθ and less net HRR compared to B20. The POME20 containing 100 ppm CuO nanoparticles used by Perumal and Ilangkumaran [125] showed a reduced BSFC to a substantial 1.0 %, reduced emissions of CO, HC, smoke and NOx by 29 %, 7.9 %, 12.8 % and 9.8 %, respectively but the BTE was improved by 4.01 % contrasted to the POME20 blend. Chandrasekaran et al. [126] outlined that the BTE had been enhanced by 2.19 % with the use of MME20 blend with 50 ppm CuO nanoparticles than that of MME20 blend at fully engine load. A reduction of up to 5.33 %, 33 % and 12.5 % were also achieved for level of HC, CO and smoke, but an increment of 3.2 % in the level of NOx was noticed. Fig. 4 summarizes the engine outputs when CuO nanoparticles are added to different biodiesel blends.

#### 3.1.4 Effect of TiO_2_ nanoadditives on biodiesel blends combustion

The experiment results of the comparative study by Gad and Jayaraj [116] revealed that blending JBD with TiO_2_ nanoparticles reduced the level of exhaust emissions with approximately 22 % for HC and 50 % for smoke emissions than that of other fuels. Kumar et al. [127] obtained an increase in the engine BTE by about 1.4 % and 3.0 % with methyl ester of orange oil (OOME) blend with 50 ppm and 100 ppm TiO_2_ nanoemulsions fuel, respectively, contrasted to neat OOME. Significant reduction was captured in the level of exhaust emissions recorded up to 24.2 % for smoke, 9.7 % for NOx, 18.4 % for CO and 16.0 % for HC when the OOME blended with 100 ppm TiO_2_ nanoemulsion fuel at the highest load. The experimental results obtained by Karthikeyan and Viswanath [128] showed that the use of TiO_2_ nanoadditives with tamanu biodiesel blends obtained from punnai seeds reduced emissions and provided better engine performance. Venu et al. [129] reported that the test results obtained for POB30 blend with 25 ppm TiO_2_ nanoparticles with EGR system application were outstanding, with a clear decrease in BSFC, and the level of HC and CO and an higher EGT compared to the conventional system without adding TiO_2_ nanoparticles.

**Fig. 5:**
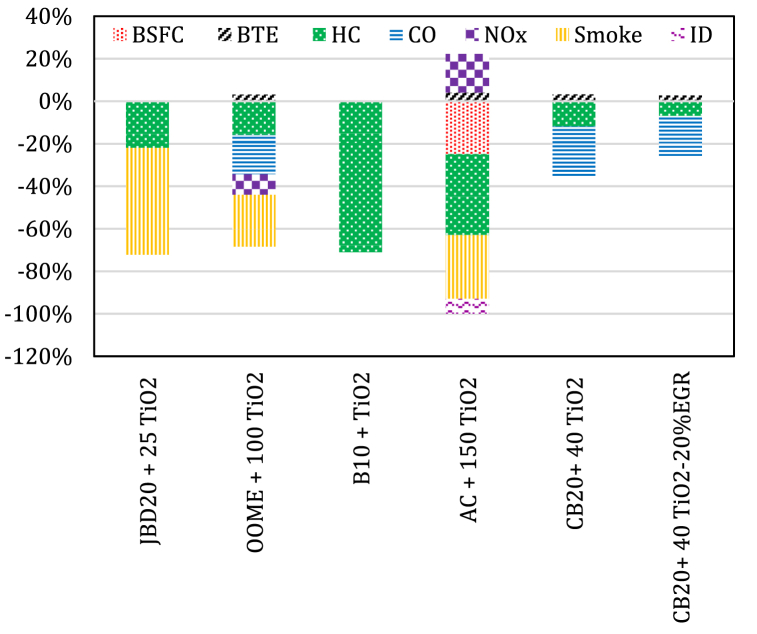
The average percent change in engine outputs for biodiesel blends with TiO_2_ nanoparticles.

Ağbulut et al. [95] reported an average reduction of 70.94 % in HC emissions for all load conditions using WCO10 blend with TiO_2_ nanoparticles. Saxena et al. [130] reported that a modified nano-fluid (MNF150) fuel consisting of 40 % Acacia concinna (AC) biodiesel, 60 % diesel, 150 ppm TiO_2_ nanoparticles and 20 ml/l isopropanol was the optimal fuel mixture, recording an increase in BTE (3–4%) and NOx emissions (5–18 %) while reducing BSFC (14–25 %), HC emission (22–38 %), smoke (20–30 %) and ID by (5–7%) compared with a pure AC biodiesel blend. Praveen et al. [100] reported that the BTE of CB20 blend with 40 ppm TiO_2_ nanoparticles without and with the application of 20 % EGR have increased by 3.1 %, 2.5 %, respectively. CO emissions got decreased by up to 23 % for the CB2040TiO_2_ fuel and increased by 14.7, 18.7 % for the CB20 + 20%EGR, CB2040TiO_2_ + 20 % EGR fuels respectively than that of CB20 blend. The level of HC emissions got decreased by 12 % for the CB2040TiO_2_ fuel sample and increased by 13 % and 7 % respectively for the CB20 + 20%EGR and CB2040TiO_2_ + 20%EGR than that of CB20 fuel sample. NOx emissions were higher for fuel CB2040TiO_2_ and lower for fuel samples CB20 + 20%EGR, CB2040TiO_2_ + 20%EGR than that of CB20 when the engine is fully loaded. Fig. 5 summarizes the engine outputs when TiO_2_ nanoparticles are added to different biodiesel blends.

### 3.2 Graphene and carbon based additives

Carbon-based nanomaterials include carbon nanotubes, graphene and its derivatives, and graphene oxide. Carbon nanotubes have lately been recognized as an additive to biodiesel to increase combustion efficiency. Multi-walled carbon nanotubes and single-walled carbon nanotubes are the two main types of carbon nanotubes.

#### 3.2.1 Effect of CNTs addition on biodiesel blends combustion

CNTs are hollow cylinders made up of carbon atoms. Their shape is graphene tubes rolled into hexagonal carbon rings with walls, predominantly formed in large bundles. The ends of CNTs are domed structures consisting of six-membered rings closed by a five-membered ring. Two types of nanotubes, including SWCNTs and MWCNTs, differ in the order of the graphene cylinders. SWCNTs only have a single graphene cylinder layer and MWCNTs have many layers (approximately 50), and they are used to improve the fuels properties [7]. Functional CNTs contain highly reactive amide groups and can react with a wide variety of chemicals. The most eye-catching features of the CNTs are their electronic, mechanical, optical and chemical properties, which have opened a path for future applications. CNTs are generally added to enhance the blend burning process and reduce its harmful products in the exhaust gas. CNTs have the ability to trap free radicals and the carbon fibers can act as an anti-knock additive. Carbon fibers can be used as sequestrants for tramp metals/tramp ions present in engine fuels to reduce the formation of insoluble complexes, resulting in fewer insoluble impurities. In addition, an increased cetane number and accelerated combustion are the resultd when CNTs are added in diesel engines.

**Fig. 6:**
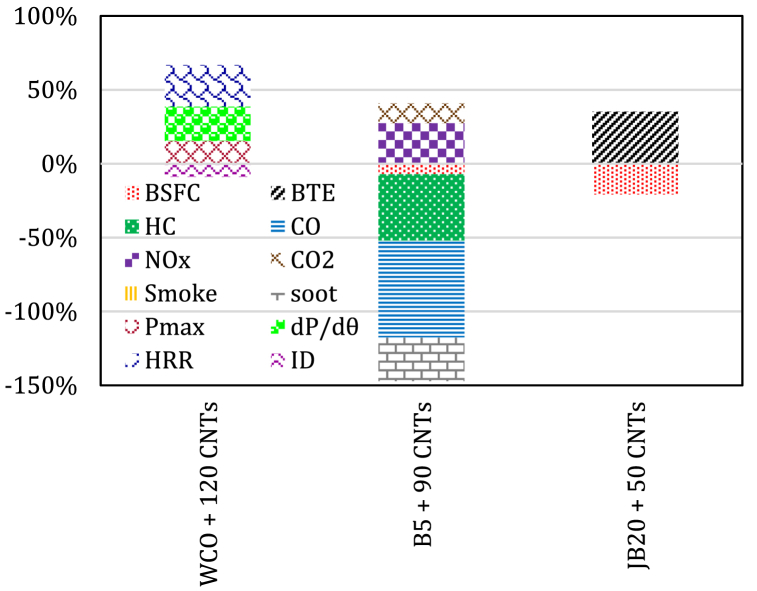
The average percent change in engine outputs for biodiesel blends with CNTs.

The results obtained by Vellaiyan [131] showed that pure SB and SB50 blends with CNTs support a shorter ID period and a net rate of HRR than that of PD. Pure SB and SB50 blend with CNTs revealed an increment in BSFC and drop in BSEC and EGT with a lower extent of the level of HC, CO, CO_2_ and smoke, excluding level of NOx emissions. Najafi [97] indicated a rise in the maximum pressure by likely 15.38 % for WCO biodiesel blend with 120 ppm CNTs compared to pure diesel. The improved fuel mixture also showed an increase in peak pressure rise and HRR by 23.33 % and 28 %, respectively, and ID was reduced by 8.98 %. Hosseini et al. [132] observed that the greatest variations of all engine outputs and combustion products occurred when a CI engine powered by biodiesel blend (B5) with 90 ppm CNTs at 2800 rpm, where an increase of up to 3.67 % for power, 8.12 % for BTE, 5.57 % for EGT, 13.18 % for CO_2_, and 27.49 % for NO_x_ was recorded compared to those of neat diesel. The levels of SFC, CO, UHC and soot got decreased by 7.12 %, 65.70 %, 44.98 %, and 29.41 %, respectively than that of diesel. Badawi et al. [133] reported that JBD20 blend with 50 ppm CNTs showed a maximum increase of about 34.9 % in BTE and a reduction of about 20.8 % in BSFC over diesel fuel. Also, it remarkably decreased the level of HC, NOx, CO and CO_2_. Fig. 6 summarizes the engine outputs when CNTs are added to different biodiesel blends.

#### 3.2.2 Effect of GO nanoadditives on biodiesel blends combustion

GO is made by the Modified Hummers Method [134] and GNP is produced by the Vapour Deposition method [135]. The manufacturing procedure of GO and GO nanoplatelets (GNPs) is very complicated, therefore, its application as conventional fuel additives is limited. Much exploration is in progress to develop affordable flow production approaches for GNPs manufacturing. The fundament graphene in GO and GNP is unreactive in nature and has high thermal equilibrium [136].

**Fig. 7:**
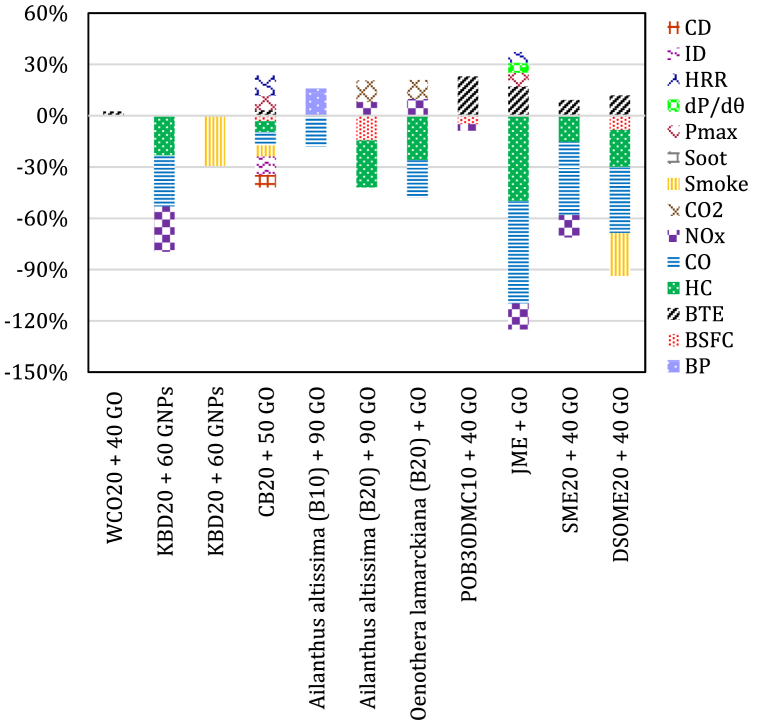
The average percent change in engine outputs for biodiesel with GO nanoparticles and GNPs.

Najafi et al. [137] reported a reduced UHCs, CO, and BSFC with a rise in the NOx level by adding 60 ppm of GO nanoparticles to biodiesel blends (B20) produced from Evening primrose (Oenothera lamarckiana), the fruit of Tree of heaven (Ailanthus altissima) and Camelina (Camelina sativa). However, 40 ppm of GO nanoparticles observed a maximum of 29.2 % smoke reductions. The maximum enhancement in BTE was obtained when 40 ppm of GO nanoparticles were added to WCO20 blend, where the BTE increased by 2.3 %. Prakash and Gowtham [138] reportedan an increase of 3.28 %, 8.21 %, and 11.85 % in BTE, P_max_, HRR when 50 ppm GO nanoparticles were added to CB20 blend. ID, combustion duration, BSFC, CO, UHCs and smoke opacity got decreased by about 10.52 %, 7.4 %, 3.2 %, 7.8 %, 6.4 %, and 6.6 % with CB20 blend with 50 ppm GO nanoparticles. Hoseini et al. [101] outlined that dispresing GO nanoparticles into Ailanthus altissima biodiesel blends enabled a crucial reduction of about 7–20 % and 15–28 % in CO and UHC emissions, respectively; nevertheless, an increment in the concentration of CO_2_ and NOx by about 6–10 % and 5–8%, respectively were noticed. The addition of B10 blend with 90 ppm GO nanoparticles cause an enhancement in brake power of approximately 15.81 % with a reduced level of CO emissions by approximately 18 % CO. The addition of B20 blend with 90 ppm GO nanoparticles caused a decrease in SFC of approximately 14.48 % with a crucial decrease in level of UHCs of approximately 27.47 %. Marginal increment in CO_2_ emissions of approximately 12.54 % and NOx emissions of approximately 7.93 % were observed in comparison to biodiesel under the same circumstances. The results obtained by Najafi et al. [81] showed that power and EGT were significantly increased using Oenothera lamarckiana biodiesel blend with GO nanoparticles. Also, reduced level of CO and UHCs emissions by around (5%–22 %) and (17%–26 %) was noticed. However, the levels of CO_2_ and NO_x_ emissions were a slightly increased by around (7%–11 %) and (4%–9%). The experimental results obtained by EL-Seesy et al. [139] reported an increase of 17 % in the BTE when GO nanoparticles are added to JME blend in diesel engine than that of pure JME blend. An increase of up to 8 % for P_max_, 6 % for dP/dθ_max_, and 6 % for the maximum HRR was also recorded. Signifcant reduction in the level of emissions reached up to 60 % for CO, 50 % for UHC and 15 % for NO_x_ was recorded. The results also showed that the 50 mg/l concentration had optimum engine performance outputs and level of emissions. Paramashivaiah et al. [140] revealed an enhancement in BTE of 9.14 %, a decrease in UHC of 15.38 %, CO by 42.85 % and NO_x_ emissions of 12.71 % with the use of simarouba methyl ester (SME20) blend with 40 ppm graphene nanoparticles. The results obtained by Soudagar et al. [141]revealed an enhancement of 11.56 % in BTE, decrease of 8.34 % for BSFC, 21.68 % for UHC, and 24.88 % for smoke and 38.662 % for CO when dairy scum oil methyl ester (DSOME20) was blended with 40 ppm GO nanoparticles. Fig. 7 summarizes the engine outputs when GO nanoparticles and GNPs are added to different biodiesel blends.

#### 3.2.3 Effect of MWCNTs on biodiesel blends characteristics

In fact, CNT is a structure of elemental carbon, that can be used as a deoxidizer to control the formation of NOx emissions during combustion as shown in equation (8). The MWCNT is known for its higher mechanical strength and superior thermal conductivity. The electrons located on the surface of carbon atoms in MWCNT make it an excellent sorbent for absorbing NO, SO_2_ and CO_2_ gases [142].

(8) $$C+2NO\to N_{2}+{CO}_{2}$$

**Fig. 8:**
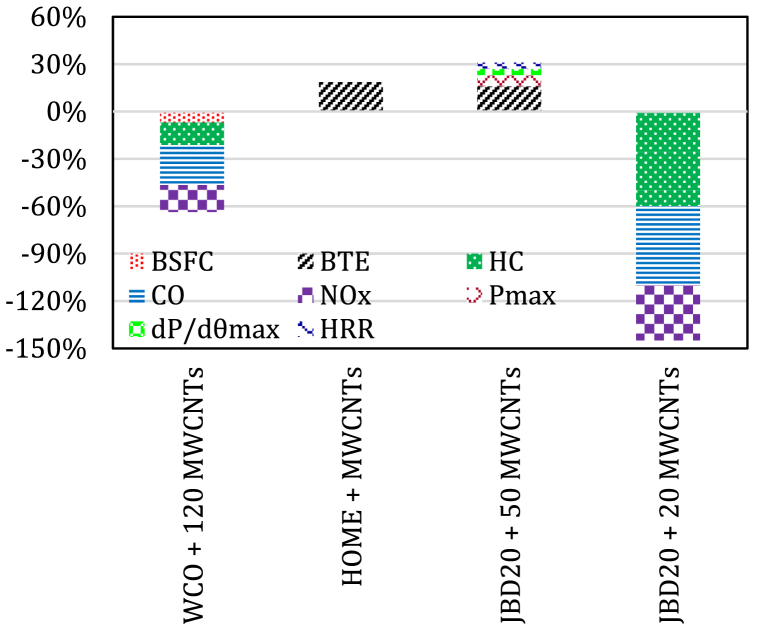
The average percent change in engine outputs for biodiesel blends with MWCNTs.

Najafi et al. [143] reported an improvement on the engine performance outputs when 120 ppm MWCNTs were added to WCO20 biodiesel blend with up to 2 % higher power and torque and 7.08 % lower BSFC than that of pure diesel. The level of CO_2_ emissions was 17.03 % higher, while it was 25.17 % lower for CO emissions than that of pure diesel. The level of UHC emissions increased by a maximum of 14.21 % and the increase in NOx emissions reached up to 25.32 % than that of pure diesel. The engine test conducted by Tomar and Kumar [144] revealed up to a 2–13 % improved BTE and up to a 5–60 % decrease in the level of emissions for Schleichera oleosa biodiesel (B20) blend with Al_2_O_3_ nanoparticles and MWCNTs. Al_2_O_3_ nanoparticles showed improved results than MWCNTs in terms of higher BTE, lower SFC and lower level of emissions than that of diesel. However, the 100 ppm MWCNT led to the maximum reduction of NO emissions due to the excellent sorbent of nitrogen oxides. EL-Seesy et al. [145] demonstrated a maximum improvement of 16 % in BTE and a decrement of 15 % in BSFC with JBD20 blend with 50 mg/l MWCNTs achieved compared to JBD20. It also provided an improvement in combustion characteristics reached up to 7 % for Pmax, 4 % for dP/dθ_max_ and 4 % for peak HRR. The exhaust emissions was significantly reduced when a concentration of 20 mg/l MWCNTs was added, The recorded decrement was up to 35 % for NOx, 50 % for CO, and 60 % of UHC. Fig. 8 summarizes the engine outputs when MWCNTs are added to different biodiesel blends.

#### 3.2.4 Effect of graphene nanopletes on biodiesel blends characteristics

**Fig. 9:**
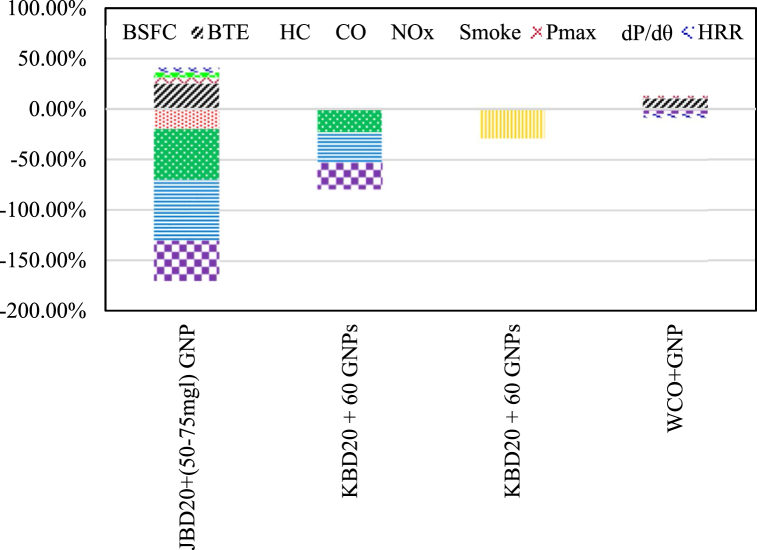
The average percent change in engine outputs for biodiesel blends with GNPs.

Graphene is familiar for its excellent physical properties and extensive technical applications. Jeyaseelan and Chacko [137] reported that adding both GO nanoparticles and GNPs to karanja biodiesel (KBD20) and WCO20 blends were attractive for better emission control of a turbocharged diesel engine. KBD20 with 60 ppm of GNPs provided lower level of emissions of CO by 30 %, HC by 23.2 % and NO by 26.3 %. Razzaq et al. [139] reported a 5.05 % maximum reduction in BSFC and 22.80 % maximum BTE with a 3.65 % reduction in the level of NOx emissions when 40 ppm GNPs and 10 % v/v dimethyl carbonate (DMC) are added to (B30) biodiesel contrasted to all other tested fuels. The effects of the addition of graphene nanoplatelets in a mixture of methyl ester of jatropha and diesel on the performance, burning process and its products in the exhaust of a CI engine have been studied experimentally by El-seesy et al. [146]. GNPs were dispersed at various dosage of 25, 50, 75 and 100 mg/l of JB20. Adding PNB at 50–75 mg/l of JB20 caused a 25 % improvement in BTE and a 20 % reduced BSFC than that of JB20. An increase by 6 % for P_max_, 5 % for dP/dθ and 5 % for HRR was reported. Additionally, the level of NOx, CO, and UHC from engine got decreased by 40 % for NOx, 60 % for CO, and 50 % for UHC at a GNP concenteration of 25–50 mg/l. The optimal enhancement in engine performance and emissions is exhibited at a concentration of 50 mg/l dosage. Sharma et al. [147] explored the influence of dispersing lti-layer additives of graphene and graphite nanoparticles in waste biodiesel from cooking oil on engine combustion and emissions. An increase by about 0.5–2.5 % in P_max_ was recorded when Multilayer graphene and graphite nanoparticle was added to the liquid fuel while HRR was reduced by1–4% when the engine is fully loaded. In addition, the level of NOx emissions got reduced by 0.7–5% with the use of multilayer graphene and graphite in the fuel mixture contrasted to that of pure diesel. Furthermore, adding 100 ppm of multilayer graphene and graphite nanoparticles into waste biodiesel increased the BTE by 8–10 % when the engine is fully loaded compared to diesel and waste biodiesel. Fig. 9 summarizes the engine outputs when GNPs are added to different biodiesel blends.

### 3.3 Effect of hybrid and tertiary nano-biodiesel blends on combustion

The results obtained by Murugesan et al. [148] revealed an enhancement in the BTE and BSFC of WCO20 blend with a mixture of Al_2_O_3_ and CeO_2_ nanoparticles were by up to 1.6 % and 8 %, respectively. In addition, a decrease in the level of emissions was recorded and reached up to 5 % for CO, 9 % for UHC, 8 % for NOx and 16 % smoke density, respectively. The results analysis exhibited an improvement in the engine outputs when 500 mg/l of Al_2_O_3_ and 500 mg/l of CeO_2_ nanoparticles are added to WCO20 blend. Prabu's experimental work [149] reported a maximum improvement of 12 % in BTE for test fuel of biodiesel B20 blend with a mixture of 30 ppm Al_2_O_3_ and 30 ppm CeO_2_ nanoparticles, followed by a 9 % enhancement in BTE for test fuel biodiesel B100 blend with a mixture of 30 ppm Al_2_O_3_ and 30 ppm CeO_2_ nanoparticles, compared to B100. NO emissions were significantly reduced for test fuel B20 blend with a mixture of 30 ppm Al_2_O_3_ and 30 ppm CeO_2_ nanoparticles with a percentage reduction of 30 %, when compared to B100. A maximum reduction of up to 60 % for CO, 44 % for UBHC and 38 % for smoke opacity was obtained for the B20 blend with a mixture of 30 ppm Al_2_O_3_ and 30 ppm CeO_2_ nanoparticles. Among the test fuels nanoparticles dispersion, B20 blend with a mixture of 30 ppm Al_2_O_3_ and 30 ppm CeO_2_ nanoparticles have significantly improved the engine outputs. Prabu and Anand [65] also reported a significant improvement in BTE for JBD blends with a mixture of 30 ppm Al_2_O_3_ and 30 ppm CeO_2_ test fuel, close to that of pure diesel. A significant reduction of 13 % in NO, 60 % in CO, 33 % in UHC and 32 % in smoke emission were observed.

The test results obtained by Hawi et al. [150] showed a P_max_ improvement of about 3.5 % when the iron-doped CeO_2_ (FeCeO_2_) nanoparticles were dispersed into the WCO. A reduction in the level of emissions reached up to 15.7 % for NOx, 15.4 % for CO, while the level of UHC kept almost same. BSFC was lower when 10 % FeCeO_2_ nanoparticles are added to WCO30 blend and the engine loaded at medium level when compared to the engine BSFC of pure diesel at high loads. Therefore, higher BTE was noted for the blend at low to medium load conditions than that of pure diesel. Karthikeyan and Prathima [151] reported that the use of doped TiO_2_ and SiO_2_ nanoparticles added to biodiesel blend (B20) produced from Botryococcus braunii algal oil in a diesel engine had shown lower CO, HC emissions while maximum NOx and CO_2_ emissions were recorded compared to diesel. Basha and Anand [73] reported a crucial enhancement in BTE and a slight decrease in the level of exhaust emissions for JBD blend with a mixture of Al_2_O_3_ nanoparticles and CNTs compared with pure biodiesel fuels. The maximum observed BTE was 28.9 % for the JBD25 blend with 25 ppm Al_2_O_3_ nanoparticles and 25 ppm CNTs, while the corresponding 24.9 % for the JBD at fully loaded engine condition. The observed smoke opacity was 57 % for the JBD blend with 25 ppm Al_2_O_3_ nanoparticles and 25 ppm CNTs, while it was 67 % for the JBD at full load. Wu et al. [152] reported that adding carbon-coated aluminium (Al@C) nanoparticles to PO10 blend could reduce the BSFC by an average of 6 % and a 6 % decrease in the level of NOx and a 19 % decrease in CO than that of PO10.

**Fig. 10:**
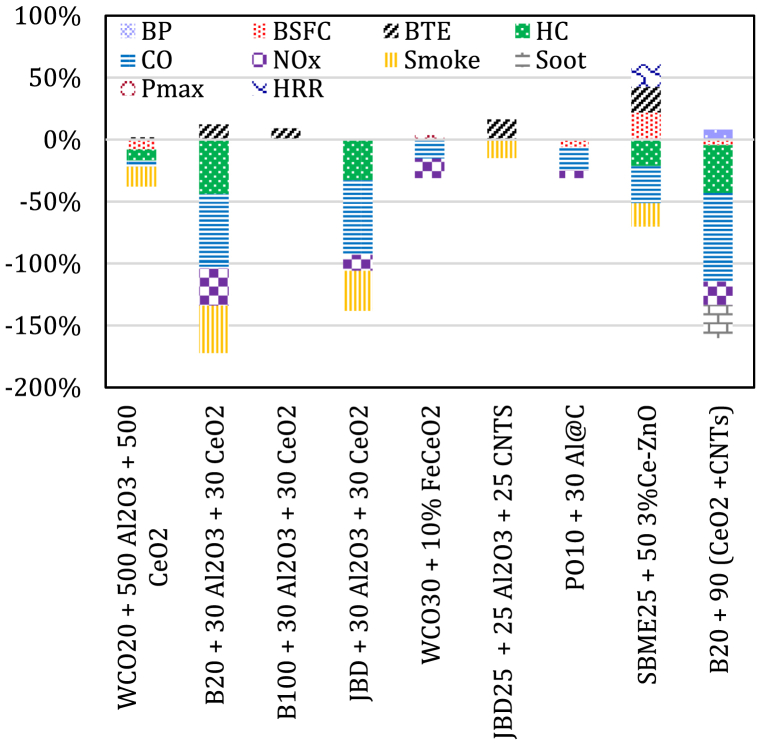
The average percent change in engine outputs for biodiesel blends with nanoparticles combinations.

Hussain et al. [153] reported an increase by 20.66 % for BTE and 18.1 % for HRR with the utilization of 50 ppm of 3 % cerium coated zinc oxide (Ce–ZnO) nanoparticles as additives in SBME25, y; BSFC decreased by up to 21.81 %; and the level of emissions decreased by 30 % for CO, 18.7 % for smoke, and 21.5 % for HC than that of SBME25 blend. However, an increased level of NOx emission was recorded when all the nanoparticle mixtures are added. Pimenidou et al. [154] recorded an increased BSFC with reduced HC, CO and NOx emissions for biodiesel blend with nanoscale cerium-zirconium-aluminium (CZA2). Mirzajanzadeh et al. [58] recorded a reduction in the level of emissionsby 18.9 % for NOx, 38.8 % for CO, 71.4 % for HC and 26.3 % for soot with the use of B20 blend with 90 ppm of hybrid nanocatalysts containing CeO_2_ on MWCNTs with amide-function compared with B20. The nanofuel blend also improved the engine's performance outputs by up to 7.81 % for power and 4.91 % for torque with 4.50 % reduction in BSFC. Fig. 10 summarizes the combinations of nanoparticles are added to different biodiesel blends.

Some nanoadditives have not been extensively studied, such as cobalt oxide, chromium oxide (Cr_2_O_3_), zirconium oxide (ZrO_2_), Fe_2_O_3_, MgO, NiO, SiO and ZnO. Jaikumar et al. [155] reported that P_max_ and net HRR improved by 18.66 % and 11.61 % at maximum load for Flaxseed oil biodiesel blend (B20) blend with 75 ppm chromium oxide (Cr_2_O_3_) nanoparticle and 1:1 dispersant (QPAN80) (B20Cr_2_O375DS1), while BTE improved by 3.62 % and BSFC decreased by 3.53 % than that of diesel. Reductions in the level of CO, UHC, NOx, and smoke reached up to 14.05 %, 12.93 %, 6.66 %, and 22.4 % respectively. Janakiraman et al. [156] reported that Garcinia gummi-gutta biodiesel (B20) blend with ZrO_2_ nanoparticles showed the lowest BTE and highest CO emissions compared to the blend with CeO_2_ and TiO_2_ nanoadditives. At peak load, B20 blend with (25 ppm ZrO_2_ nanoparticles reduced BSEC than B100 by 22.11 %, HC emissions than B20 by 5.64 %. At partial engine loading of 25 %, the level of CO_2_ emission from B20 + ZrO_2_ (25 ppm) were observed to be better than B20 mixture by 12.95 %. Muthusamy et al. [42] concluded that the POME20 biodiesel blend with Fe_3_O_4_ nanoparticles enhanced the performance outputs of the CI engine and significantly decreased the CO, HC and smoke products, while the NOx emissions were higher compared to B20. The experimental results obtained by Jumaa and Mashkour [157] showed that adding Fe_2_O_3_ nanoadditives to biodiesel blends enhanced BTE by 15.05 % and decreased BSFC by 10.73 %. In addition, the level of exhaust emissions, and decreased by up to 62.5 % for CO, 63.01 % for HC and 28.9 % for smoke density, but NOx and particulate matter increased by 16.19 %, 15.30 %, respectively, than that of diesel.

Abdallah et al. [158] revealed that a biodiesel (B30) blend with 50 ppm MgO nanoparticles showed a 19 % reduction in BSFC than that of diesel. The highest BTE increase has been identified as 23.7 %, with a reduction of up to 7 % for NOx emissions. Ozgur et al. [159] outlined that the level of NOx and CO products were decreased and engine performance outputs were improved when MgO nanoparticles are added to biodiesel blend. Fig. 10 summarizes the engine outputs when MgO nanoadditives are added to different biodiesel blends. Srinidhi et al. [160] revealed that advancement of fuel injection timing with NiO nanoparticles to Azadirachta indica biodiesel (B25) blend base fuel increased the BTE by 6.3 %. Average reductions in BSFC were reported when NiO nanoparticles are added to B25 blend at the injection timing of 27°bTDC and reached up to 5.29 % for the dosage of 25 ppm, 6.91 % for 50 ppm, 7.13 % for 75 ppm and 7.86 % for 100 ppm for with. The results obtained in different experiment done by Srinidhi et al. [161] revealed that using 75 ppm NiO nanoparticles in BN25 blend reduced the amount of thermal NO_X_ by 6.2 % and BSFC by 1.8 % with improved BTE by 2.9 % compared with the case without the nanoparticles. Ağbulut et al. [95] proved an enhancement in the burning process quality of a single-cylinder CI engine when SiO_2_ nanoparticles are added to WCO10 blend. An average reduction in the level of HC emissions reached up to 80.98 % was obtained for all load conditions. Gavhane et al. [162] revealed an increase of around 3.48–9.12 % in BTE and a reduction of around 5.81–11.58 % in BSFC when SiO_2_ nanoparticles are added to Soybean Methyl Easter (SBME25). The level of CO, HC and smoke got decreased by 1.9–17.5 %, 20.56–27.5 % and 10.16–23.54 %, respectively than that of SBME25 blends. The results obtained by Ozgur et al. [159] showed that NO_X_ and CO emissions from the engine exhaust were reduced and performance was slightly improved by using biodiesel blends with SiO_2_ nanoparticles.

**Fig. 11:**
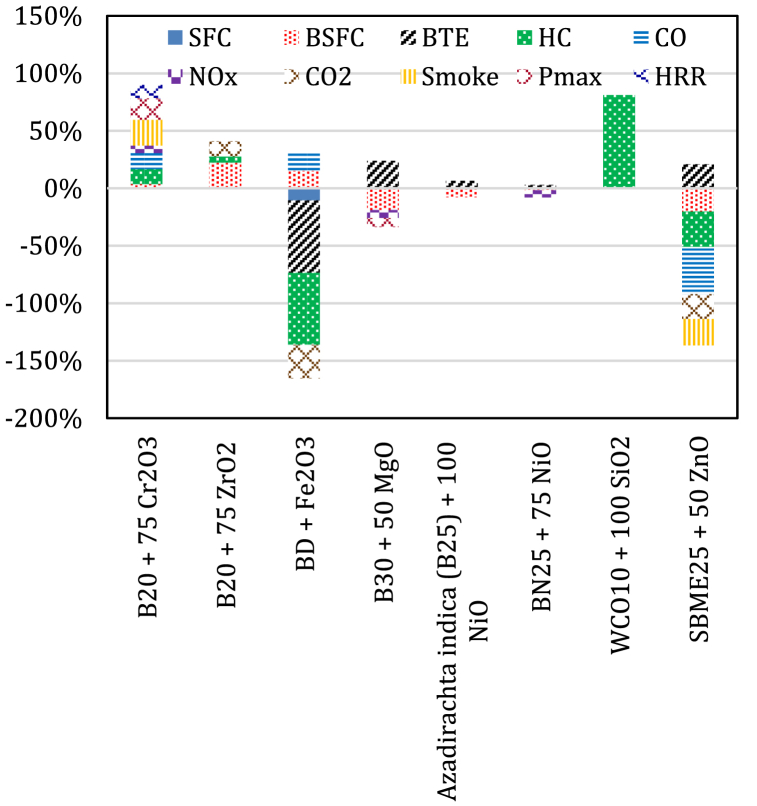
The average percent change in engine outputs for biodiesel blends with different nanoparticles.

The test results obtained by Karthikeyan et al. [77] indicated an increase in BTE, EGT, and the level of NOx and CO_2_ emissions with a reduction in BSFC and the level of CO, HC and smoke emissions when a proportion of ZnO nanoparticles were added to GSOME blends. Abdallah et al. [158] recorded a lower nitrogen oxide emission concentration of 385 ppm for the B30 biodiesel blend with 50 ppm ZnO nanoparticles at full load compared to the pure B30 blend. Gavhane et al. [163] reported an increase of up to 20.59 % in BTE with a decrease of 20.37 % in BSFC when 50 ppm ZnO nanoparticles were added to SBME25 blend at a compression ratio of 21.5 than the one of SBME25 blend. The level of combustion products got decreased by up to 30.83 % for HC, 41.08 % for CO, 22.54 % for smoke and 21.66 % for CO_2_, while the level of NOx emissions showed a slight increase. The results obtained by Karthikeyan et al. [79] reported an increased BTE and significantly reduced BSFC, CO, HC and smoke opacity at all engine loads when ZnO nanoparticles were added to Pomolion Stearin Wax Oil Methyl Ester (PSWME), while NOx emissions had no significant difference. Fig. 11 summarizes the engine outputs when different nanoparticles are added to different biodiesel blends.

## 4 Conclusions

Based on the literature discussed in this article, it can be concluded that the application of a mixture of metallic energetic particles at the nanoscale in biodiesel blends is an interesting concept that has not yet explored full potential. These nanoparticles have the advantages that make it feasible to be added into the convetional fuels and produce such types of nanofuels that can be a commercial substitute. The mentioned research have revealed that the combustion of CI engines by adding formulated nanoscale at different quantities to biodiesel is conductive to reducing BSFC, improving BTE and further improving emissions of CO, UHC and NO_x_. This can overcome the drawbacks of operating CI engines with pure biodiesel blend fuels. However, there is no information for studies on the use of certain types and dose levels of nanoadditives in the blends to run engines. Given the limited number of research studies on the optimal nanoadditives combinations and their impact on the blend quality, there is a need to investigate the engine outputs when these nanoadditives are added to blends. From the discussion of the influence of nano-additives on fuel properties, very promising engine outputs were seen when nanoadditives are added into blended fuels. However, nanoparticle additives are not sensitive beyond a certain concentration level and it is necessary to determine the optimal dosage for each nanoparticle. In addition, the influence of nano-biodiesel on engine outputs, emissions and combustion characteristics of traditional fuels remain a major problem limiting their applications. As per the review, future studies can focus on hybrid nanoparticles, which are composed of two or more nanoparticles.

## Data availability statement

Data included in article/supp. material/referenced in article.

## CRediT authorship contribution statement

**Bahaaddein K.M. Mahgoub:** Conceptualization, Formal analysis, Investigation, Methodology, Resources, Writing – original draft, Writing – review & editing.

## Declaration of competing interest

The authors declare no competing interests.
